# Participatory research with youth with disabilities: Experiences from sub-Saharan Africa

**DOI:** 10.4102/ajod.v13i0.1491

**Published:** 2024-10-21

**Authors:** Femke Bannink Mbazzi, Shaffa Hameed, John K. Ganle, Tom Shakespeare, Sarah Polack

**Affiliations:** 1 Disability Research Group, MRC/UVRI & LSHTM Uganda Research Group, Entebbe, Uganda; 2 International Centre for Evidence in Disability, London School of Hygiene and Tropical Medicine, London, United Kingdom; 3 School of Public Health, University of Ghana, Accra, Ghana

**Keywords:** participatory research, youth, disability, employment, education, Africa

## Abstract

**Background:**

Disability inclusive youth research, involving youth with disabilities in the design, implementation and dissemination of study data, is still limited in Africa.

**Objectives:**

To describe and reflect on the experiences of involving youth with disabilities in an exploratory research study, focused on disability-inclusive education and employment in 7 African countries.

**Method:**

12 youths with different impairments, aged 18 to 35, were employed as researchers in Ethiopia, Ghana, Kenya, Nigeria, Rwanda, Senegal and Uganda. Youth researchers contributed to the data collection and analysis of interviews with 210 youth with disabilities. 24 youth advisors with disabilities formed two youth advisory groups (YAG) of 12 advisors each in the regional hub countries Ghana and Uganda. The YAGs met 4 times during the project and contributed to the study design, data collection, data analysis and dissemination activities. In addition, 4 workshops were held with the Ugandan YAG to develop a participatory film.

**Results:**

Together with the youth participants, we reflected on the experiences of involving youth with disabilities and conducting research with, by and on youth with disabilities. We highlighted ethics and safeguarding, recruitment and representation, exploring experiences and data quality, participatory dissemination, accessibility, capacity building and networking as key areas of consideration and benefit in this project.

**Conclusion:**

Participatory research with youth with disabilities is feasible, enriching, and key to inclusive research that informs education and employment policy and practices.

**Contribution:**

Lessons learned from youth involvement in a disability inclusive research programme, focused on education and employment in 7 African countries.

## Introduction

Globally, young persons with disabilities are left behind in access to health care, education, social participation and employment (Kuper et al. 2014; World Health Organization 2022). Youth with disabilities are less likely to attend school and those who do attend have lower educational attainment compared to their without disability peers (United Nations Children’s Fund 2021). The risk of unemployment for youth with disabilities is high, and even if they are employed, they are more likely to have irregular employment and lower earnings (Banks, Kuper & Polack 2017; Mactaggart et al. 2018). The coronavirus disease 2019 (COVID-19) pandemic has further exacerbated the exclusion of youth with disabilities (Shakespeare, Ndagire & Seketi 2021).

Over 70% of the population of sub-Saharan Africa is under 30 years old; approximately 12% of them have a disability (United Nations 2022). To understand the experiences of young people with disabilities and develop contextually appropriate and effective strategies to promote participation, youth with disabilities need to be included throughout the research process (Bailie et al. 2023; Kuper et al. 2014; Lundy, Mcevoy & Byrne 2011).

Participatory research with youth with disabilities in sub-Saharan Africa must be underpinned by respect, equitable partnerships, barrier removal and the use of appropriate tools (Chappell et al. 2014; Kuper et al. 2021; Wickenden & Kembhavi-Tam 2014). This requires a shift in power from researchers from high-income countries to people with disabilities and researchers in low-income countries as well as researcher–subject power dynamics (Kuper et al. 2021; Njelesani et al. 2022), consideration of African research methods (Mkabela 2005; Owusu-Ansah & Mji 2013) and youth-friendly research processes (Chappell et al. 2014; Njelesani et al. 2022; Wickenden & Kembhavi-Tam 2014). Research WITH and BY youth with disabilities rather than ON youth with disabilities only, is key to avoid a tokenistic approach (Kuper et al. 2021; Njelesani et al. 2022).

In the ‘Young Africa Works: Disability Inclusive Research Partnership’ project, we designed a participatory research study with youth with disabilities in Ethiopia, Ghana, Kenya, Nigeria, Rwanda, Senegal and Uganda. We recruited youth with disabilities as researchers, created youth advisory groups (YAGs) and asked youth participants with disabilities to co-create a participatory film to share study findings.

Participatory film has been used as a way to give voice to marginalised groups, in which participants use cameras to document, explore and engage with their environment and create a film that communicates information, reveals hidden information and stimulates community action (Gubrium, Harper & Otañez 2015). Participatory filmmaking is a co-creative flexible process, which uses preparatory, participatory and action phases (Lorenz & Kolb 2009). Steps include brainstorming ideas, getting to know the equipment, storyboarding, shooting the films, watching, and reflecting on the films and the process and dissemination (Benjamin-Thomas et al. 2019). Important aspects of participatory film in our research context are the interdependence between the youth and their family and communities, power relations and ethical considerations (Bannink Mbazzi et al. 2024).

In this article, we describe our experiences and reflections on a participatory research project working with youth with disabilities as researchers, as advisory members (conducting research WITH) and as study participants (conducting research ON) to explore the experiences of youth with disabilities in education and employment.

## The ‘Young Africa Works: Disability Inclusive Research Partnership’ project

The ‘Young Africa Works: Disability Inclusive Research Partnership’ project had three different levels of representation of youth with disabilities to ensure inclusion.

### Identifying and recruiting youth with disabilities as co-researchers

Firstly, a total number of 12 research team members with disabilities, from the level of team leader to research assistant were recruited in the seven countries. In the advertisement, which was made accessible for screen readers and circulated in networks of organisations of persons with disabilities through traditional methods such as newspaper adverts as well as social media, it was made clear that the project wished to recruit youth with disabilities as co-researchers. During the selection process, attention was paid to ensure barrier removal and reasonable accommodations were provided. The selected youth were offered reasonable accommodations at work. They received training on research ethics and study processes and were paid the organisation’s salary rate for their research position for the duration of employment (on average 1 year). The researchers interviewed approximately 30 youth with disabilities per country. We ensured that each research team had at least one or more researchers with a disability.

### Establishing youth advisory groups with youth with disabilities

Secondly, two YAGs were created in Ghana and Uganda, each consisting of 12 female and male youth with different impairments under 35 years. Youth advisory groups were established in collaboration with organisations of persons with disabilities in the respective countries with support from national disability organisations and networks of persons with disabilities. To ensure representation, organisations representing persons with different impairments were asked to nominate youth who could contribute to the advisory groups. Based on individual discussions and the availability of each of the proposed youth, a selection was made in each hub to ensure representation of gender and impairment groups. The groups anchored the East and West African research hubs for the multi-country programme. The YAG advised the study team at inception, during implementation and at dissemination on content and involvement of youth with disabilities and they co-led dissemination activities. In addition, YAG members were provided with training which they expressed the need for as advisory team members. For example, the Uganda group was interested in learning more about research ethics and received training on the same. The group in Uganda had six female and six male members with different impairments, including spina bifida, spinal cord injury, cerebral palsy, hearing impairment, visual impairment, albinism, chronic mental health conditions and Down Syndrome. The Ghanaian group consisted of five male and six female members with different impairments, including hearing impairment, spina bifida, spinal cord injury, visual impairment and albinism. The YAGs met on a quarterly basis. Youth advisory group members received remuneration as per national guidelines for advisory committees. Reasonable accommodations were provided and budgeted for in the project. The youth ensured that the Young Africa Works study was conducted WITH youth with disabilities and ensured dissemination was carried out BY youth with disabilities.

### Involving youth with disabilities as research participants

Thirdly, a group of 210 youth with disabilities was purposively selected as study participants in the seven countries, taking into account impairment type, age, gender, rural and urban locations, education and employment status with the aim to ensure an equitable representation. Similar to the selection of the youth researchers and advisors, we approached existing networks of persons with disabilities, as well as local leaders, rehabilitation centres, schools and employers to identify a wide range of participants. The research teams interviewed participants about their experiences in education and employment. While the research was mostly conducted ON 210 youth, it was conducted BY research teams that included researchers with disabilities. Of the 210 youth, 10 purposively selected youth participants from Ghana and Uganda were asked to participate in the participatory filmmaking process together with the YAG members, contributing to research WITH youth.

We reflected on the youth participation during team meetings with research staff in each of the two hubs, as well as the two YAGs in study advisory meetings. We also asked the youth who contributed to the participatory filmmaking to reflect on their participation in an evaluation meeting at the end of the project. The reflections were guided by questions about the barriers and facilitators to youth involvement in the project and benefits that were derived from youth involvement. The analysis process was a reflexive and informal process of discussions between the research teams and YAG members. It started from reflections which were made throughout the study and were woven into ongoing study activities such as regular team meetings, data analysis workshops and YAG meetings. Written reports of the discussions, study and advisory meetings, as well as individual country reports on the interview data from the 210 youths were reviewed by two members of the research team. They drafted a list of considerations and themes around participation and shared this with the youth advisory members and young research staff members for feedback. The key considerations and benefits included ethics and safeguarding, recruitment and representation, exploring experiences and data quality, participation in dissemination, accessibility and capacity building and networking, further discussed in the next section.

Reasonable accommodations were provided for research staff, YAG members and research participants where needed, and included sign language interpretation, provision of braille prints, screen readers, easy-read and large prints, and allowances for personal aids. Participants and persons providing aid received reimbursement for their time as well as a transport refund where meetings took place away from their home locations, following national guidelines for compensation.

## Considerations and benefits of youth involvement

### Ethical considerations

A key consideration in conducting research on youth with disabilities is the ethics around meaningful and safe involvement. For the Young Africa Works: Disability Inclusion Partnership study, ethical clearance to conduct this study was obtained from Ghana Health Service Ethics Review Committee (No. GHS-ERC: 009/08/21), Addis Ababa University Institutional Review Board (No. 006/22/SPH), Uganda Virus Research Institute Ethics Committee (No. GC/127/867), Kenyatta National Hospital - University of Nairobi Ethics Research Committee (No. P7/01/2022), National Health Research Ethics Committee of Nigeria (No. NHREC/01/01/2007), University of Rwanda, College of Medicine and Health Sciences (No. 43/CMHS IRB/2022), Global Research and Advocacy Group, National Ethics Committee for Health Research (No. SEN22/51) and the London School of Hygiene and Tropical Medicine (No. 26513). All participants gave informed consent to participate in the study. Youth who participated in the YAGs and the participatory film specifically agreed to the photo and film activities and sharing of outcomes in the public domain. Where required, consent of a parent or guardian was received on behalf of youth with cognitive or communication impairments, while the youth gave consent to participate. The filmmakers obtained permission from the relevant authorities to film in public spaces, following Ugandan and Ghanaian local policies and guidelines.

A safeguarding policy was developed for the project and all research staff were trained. Each country had specific referral pathways in place, with a list of service providers that could be consulted in case of a safeguarding concern. In addition, debriefing meetings were held within the country teams to allow researchers to reflect on their experiences during the interview data collection. The YAG members were asked to carefully reflect on their emotional well-being while participating in the meetings and more specifically the participatory filmmaking.

#### Recruitment and representation

Participant recruitment involved organisations of persons with disabilities, specialised health and education services for persons with disabilities, and contacts of YAG members, as well as snowballing. Having youth with disabilities contribute to the recruitment of study participants, enabled higher levels of representation of persons with different impairments. For example, in Ghana, a young woman with albinism supported the recruitment of participants. As participants trusted her, she was able to invite individuals who may have been hard to find and recruit if approached by other researchers.

Youth with different types of disabilities, gender, and age with experiences in secondary and tertiary education as well as employment such as agriculture, tourism, information technology (IT) and other sectors were selected to participate by the YAGs. Youth with disabilities participating in the filmmaking reflected that the diversity of the YAG ensured they made a film that was representative of a large group of youth with disabilities.

There were limitations to the participant recruitment of youth with disabilities. For example, participants suggested by Organisations of Persons with Disabilities (OPD) and other organisations tended to be highly educated, come from urban areas and do not represent the most marginalised groups of youth with disabilities, even if they come from low-income households. In addition, some youth with impairments such as those with severe cognitive impairments and others who are deaf-blind, were not included. Youth representatives felt that it would be important to identify strategies to include more marginalised youth in future programmes. These could include purposive selection and snowballing through rural community networks, seeking out persons with invisible impairments such as autism spectrum disorder, persons who are both deaf and blind, and persons with more severe neurological and speech impairments.

## Exploring experiences and data quality

Having young persons with disabilities involved in conducting interviews with youth with disabilities gives in-depth information which explores disability experiences at a different level. For example, a young researcher with a hearing impairment conducted an interview with a youth with a hearing impairment and was able to engage and delve into more detail in the interview than a researcher with a sign language interpreter. Some of the study participants mentioned that they felt at ease and could open up more as they felt their peer youth with a disability would understand their experiences better.

Youth participating in the interviews and film process mentioned that sharing their stories at times made them feel emotional. At the same time, their direct involvement in the film and continuous re-telling of their stories in the filmmaking process also allowed for genuine and in-depth sharing of lived experiences. The youth mentioned feeling heard and glad they were given a space to share their perspectives.

### Participation in dissemination

As part of the dissemination strategy, we co-created a participatory film with the YAGs, 10 study participants, and the research teams in Ghana and Uganda. In the first workshop, we worked with the existing YAGs and research teams to brainstorm about how best we could create a film that would narrate the experiences of youth with disabilities in education and employment in Africa, based on the findings from the interviews. In the second workshop, we identified and prioritised the focus of the narrative and trained youth on storyboarding and camera use. The youth identified key participants for the film and started filming different youth stories both in Ghana and Uganda. Rough footage was shared at the third workshop for feedback. In the fourth workshop, the semi-final film was shared. The groups discussed how to make the films as accessible as possible to all, and worked on captioning, audio narratives, music and text.

Youth advisory group members were also a central part of the final project workshop attended by representatives from all seven countries (including researchers, OPDs, non-governmental organisations [NGOs] and policymakers). Youth advisory group representatives presented their lived experiences, narrated the development of and presented the final version of the film and shared their experiences of making this. Research team members with disabilities additionally gave oral presentations at the workshop. The research teams in each country also shared project findings and the film with local stakeholders in in-country dissemination meetings to elicit discussion and action. In addition, a delegation of the Uganda YAG presented findings and the film at the AfriNEAD conference in South Africa, and the end of the project webinar. It was also shared with Mastercard Foundation staff in an online capacity-building workshop.

### Barrier removal and accessibility

The presence of research team members with disabilities at the various research organisations resulted in some barrier removal and accessibility initiatives. For example, the Ugandan partner organisation in the project built ramps and negotiated the use of accessible cars for the research staff with physical impairments. They also marked some entrances to enhance visibility for persons with visual impairments.

The making of the film resulted in inclusive action in some of the youth’s education and employment institutions. For example, a youth representative in Uganda who is a university student mentioned that his campus made immediate changes to the accessibility of buildings after he was filmed on campus as part of participatory filmmaking.

### Capacity building and networking

Participation of youth in the study teams, either through direct involvement in data collection or in the youth advisory, built the capacity of youth in research and advocacy. The youth mentioned to have developed research, filmmaking and presentation skills.

The youth advisory members mentioned the project had supported networking between research organisations and organisations of persons with disabilities, opened up opportunities for future disability research, and expanded relationships between youth organisations from different organisations of persons with disabilities. For example, the Ugandan YAG had a youth representative from the National Youth Association for the Deaf. This youth was able to mobilise a large group of Uganda’s deaf community for the study dissemination events and created further linkages between the research organisation and the Ugandan National Association for the Deaf.

Four of the Ugandan YAG members were selected for an internship programme at two research institutions in Uganda after their participation. Another five of them were asked to co-facilitate disability inclusion training for the project funders’ staff.

## Discussion

In this article, we described the ‘Young Africa Works: Disability Inclusive Research Partnership’ project and reflected on the experiences of involving youth with disabilities and conducting research with, by and on youth with disabilities. We discussed our experiences in ethics and safeguarding, recruitment and representation, exploring experiences and data quality, participatory dissemination, barrier removal and accessibility, capacity building and networking in the Young Africa Works: Disability Inclusion Research Partnership. In our project, we conducted research BY and WITH persons with disabilities with barrier removal, reasonable accommodations and intentional inclusion of youth with disabilities at all stages in the research project. By involving youth with disabilities as researchers and in YAGs, we were able to access a varied group of study participants, collect and share in-depth lived experiences, offer career development and training opportunities for those involved, and advocate for inclusion of youth with disabilities in education and employment.

Our approach tried to ensure culturally relevant and inclusive disability research (Owusu-Ansah & Mji 2013), with youth with disabilities as ‘significant participants’ and ‘equals’ in the research and decision-making process, following an Afrocentric approach (Mkabela 2005; Owusu-Ansah & Mji 2013). There is no structured framework or evaluation matrix for participatory research in the Afrocentric approach (Owusu-Ansah & Mji 2013), and a limitation of this article is that we only used a descriptive and informal approach to reflect on the process. Future studies could consider structured evaluation approaches and evaluation frameworks such as the Research Quality Plus for Co-Production (McLean et al. 2022) adapted to the local context in discussion with youth participants.

## Conclusion

Participatory research with and by youth with disabilities is feasible and key to inclusive research that informs education and employment policy and practices. Further participatory research studies rooted in African disability discourse with youth with disabilities are needed to establish culturally relevant evaluation practices and guidelines.
